# Comparative effects of post isometric relaxation technique and Bowen’s therapy on pain, range of motion and function in patients with temporomandibular joint disorder

**DOI:** 10.1186/s12903-024-04440-1

**Published:** 2024-06-12

**Authors:** Sunaina Javed, Muhammad Salman Bashir, Aqib Mehmood, Rabiya Noor, Mehwish Ikram, Ghazal Hussain

**Affiliations:** 1 Faculty of Rehabilitation and Allied Health Sciences, Riphah International University, Lahore, Pakistan; 2 School of Health Sciences, University of Management and Technology, Lahore, Pakistan; 3 AM Physiotherapy Clinic, Lahore, Pakistan

**Keywords:** Temporomandibular disorder, Joint pain, Range of motion

## Abstract

**Background:**

The most common cause of mouth and facial pain is a temporomandibular joint disorder, which affects the patient’s quality of life and interferes with their ability to perform daily tasks.

**Objective:**

The purpose was to compare the effects of the Post-Isometric Relaxation Technique and Bowen’s Therapy on pain, range of motion and functional activity in patients with temporomandibular joint disorders.

**Methods:**

This study was a randomized clinical trial. A total of 24 participants were randomly allocated into two groups using the lottery method. Baseline treatment was the same (ultrasound and tapping) in both groups. Group 1 (12 participants) was treated with a post-isometric relaxation technique, and Group 2 (12 participants) with Bowen’s therapy for two sessions per week (total duration of 4 weeks). Outcome measures were the Numeric Pain Rating Scale, Maximal mouth opening inter-incisal rural and jaw functional limitation scale-20. SPSS version 25 was used for statistical analysis.

**Results:**

A significant improvement in pain, range of motions and functional activities in the post-isometric group showed significant results (*p* < 0.05) as compared to Bowen’s group (independent t-test). However, within-group comparison (paired t-test), both groups showed significant results (*p* < 0.05).

**Conclusion:**

This study concluded that post-isometric relaxation was more effective in terms of pain, range of motions for mouth opening, lateral deviations and functional activity of temporomandibular joint disorder patients. However, both groups showed clinical results according to minimal clinical difference values.

**Trial registry number:**

The trial is registered under ClinicalTrials.govt with reference no. ID: NCT05392049 registered on 26/05/2022.

## Introduction

The term “temporomandibular disorders” (TMDs) refers to a variety of pathological conditions that affect the masticatory muscles, temporomandibular joint, and related structures. These conditions are characterized by pain in the temporomandibular joint (TMJ), pain in the muscles of mastication, a restriction or deviation in the range of motion of the mandible, and TMJ noises like clicking, popping, and crepitus when the mandible is functioning [1, 2]. The second most frequent cause of pain in the mouth and face is temporomandibular disorders (TMDs). Between 5 and 12% of the adult population worldwide is thought to be affected, with women at least twice as affected as men [3]. Women commonly suffer from TMDs and 45.3% of patients with TMDs have myofascial pain problems. Pain, restrictions in mouth opening, and jaw range of motions are the three most common signs of TMDs muscle-related dysfunction [4].

TMDs represent the “third” category of dental illness, it is appropriate to see them as a condition that affects the population (after dental caries and degenerative diseases). TMDs symptoms are more prevalent in females. A link was found between TMDs symptoms and oral habits, including bruxism and uneasiness in adolescents, as well as sadness and anxiety. Other studies have reiterated the importance of stress levels and sleep quality as coexisting factors in TMDs [5]. TMJ and masticatory muscle pain, limited jaw movement, popping, clicking, and grinding noises, as well as painful jaw functions are some symptoms (yawing, swallowing, chewing, biting, grinding) [6]. The causes of TMDs are multifaceted, with biomechanical, neuromuscular, psychological, and emotional factors playing a significant role [7]. One of the most widespread symptoms of TMDs is pain, which can affect the ears, eyes, and throat, causing significant neck pain and headaches as well as impaired functioning and mandibular movement abnormalities [8]. Ninety (90) percent of TMDs patients report having trouble sleeping, which is a common problem [9].

Clinical diagnosis of TMDs is done by using the Temporomandibular Joint Disorder Research Diagnostic Criteria [10–13]. The TMJ Pain Special Interest Group and the International Research DC/TMD Consortium Network developed the Diagnostic Criteria for TMDs (DC/TMD) [11, 12]. Pharmaceutical therapy, oral appliances, occlusal splints, and TMJ surgery are used as non-conservative approaches in TMDs patients. Physiotherapy, exercise, self-care, dietary changes, awareness of aggravating symptoms, and patient education are used as conservative treatments. Ultrasound enhances deep tissue membrane permeability, and blood flow, and promotes healing. The heating effect of ultrasound also reduces spasm [14]. In the type of Muscle Energy Techniques (METs) techniques, the most beneficial and relaxation technique is post-isometric relaxation (PIR) [15]. The PIR technique, which focuses primarily on neurophysiological inhibitory relaxation mechanisms (e.g. autogenic inhibition), allows for the immediate reduction of muscle tension following isometric contraction and the reduction of pain perception related to the muscular system [16].

A form of manual therapy called Bowen’s Therapy uses a special technique to manipulate soft tissues. During a Bowen’s Therapy session, the thumbs and fingers are used to apply a pattern of Bowen transitions, light pressure movements are applied to specific areas on muscle fibres, ligaments, tendons, and connective tissue. The movements are separated by 2 to 5-minute breaks to give the body time to recover [17]. It uses a soft, calming technique rather than forceful manipulation and is effective for both acute and chronic conditions [18]. Bowen’s therapy movements are non-invasive and gentle. By accelerating the flow of energy, Bowen’s therapy encourages the body to heal itself. Moving in a positive direction can encourage the flow of positive energy, whereas moving in a negative direction can impede it. It’s critical to realize that Bowen’s method is not direct energy work but rather a physical method of controlling the body’s energy flow. Bowen’s method is secure and effective for treating conditions and encourages healing by re-establishing balance within the body [19].

There are few studies conducted on the clinical effectiveness of Bowen’s Therapy. The effectiveness of Bowen’s Therapy in clinical populations has been studied in a few trials [17, 19, 20]. The effects of Bowen’s therapy have not been addressed yet in Temporomandibular (TMJ) disorders although Bowen’s therapy is a very effective technique in pain, relaxation and release of muscle tension, however, it has very limited research evidence available. Therefore, this study compared how Bowen’s therapy and post-isometric relaxation techniques affected individuals with TMJ disorder’s pain, range of motion, and function. This study adds new regimes for TMJ disorder and opens the horizon for clinicians. This study may positively affect the quality of life for TMDs patients and may add to the knowledge of the physiotherapy community and explore the new physiotherapy protocol for TMDs patients’ betterment. It was hypothesized that there is a difference between the effects of Bowen’s therapy and Post-Isometric Relaxation Technique on pain, range of motion and function in patients with TMJ Disorder.

## Methodology

The study was a randomized clinical trial (parallel assignment). The research was conducted in Riphah Rehabilitation Centre after approval from the ethical research committee of Riphah International University (REC/RCR & AHS/22/0106). The trial is registered under ClinicalTrials.govt with reference no. ID: NCT05392049 on date 26/05/2022. Sample size 24 was calculated by using G power version 3.1.9.4 software with a 5% margin of error and 0.80 power of study and effect size 1.40, the variable used for this purpose was pain from a previous study and a 20% attrition rate was added [21]. A purposive sampling technique was used for data collection. Inclusion criteria consisted of male and female participants with the age group of 20–50 years and jaw joint restrictions (limited range of motions) with clicking sounds. A complete clinical assessment was done; the patient’s clicking sound was evaluated by hand palpation on TMJ during mouth opening, closing, and (RT & LT) deviations. Participants with fractures of the jaw or TMJ and other systemic disorders were excluded from the study.

Written informed consent forms were taken from all the participants. All the participants were asked to draw a card (1 and 2) from a box. Those who selected 1 and 2 were allocated into two groups accordingly. It was a single-blinded study and the outcome assessor was blinded in this trial.

In Group 1, 12 participants were allocated and received post-isometric relaxation while 12 participants were in Group 2 received Bowen’s therapy.

### Group 1 (post-isometric relaxation technique)

The patient was in a supine lying position on the couch, the neck was supported on a comfortable pillow, and the jaw was in a resting/actual resting position. Ultrasound therapy over TMJ (Mode 1:1, Frequency 3 MHz, Intensity 1.5 W/cm2, Duration 5 min and Dosage 2/week) was done by a therapist. The therapist has applied post-isometric relaxation technique, exercises for mouth open, close, right and left lateral deviations (strengthen the weakened muscles, stretch the shortened muscles) with dosage 5 times per session, hold and 5-second rest and 2 times per week for 4 weeks as a standard treatment [22]. After the end of the treatment session taping was applied on the TMJ (y shape for improved range of motion) [23] and patient education and self-assessment regarding the symptoms were conducted.

### Group 2 (Bowen’s therapy)

The patient was in a supine lying position on the couch, her neck was supported by the comfortable pillow, and her jaw was in resting/actual resting position. Ultrasound therapy over TMJ (Mode 1:1, Frequency 3 MHz, Intensity 1.5 W/cm2, duration 5 min and Dosage 2/week) was done by therapist [21]. The therapist was sitting on a comfortable chair and applied the Bowens technique on TMJ and masticators, temporalis muscles. Two to three repetitions in 1 set and 4-to 5 sets in each session apply as a standard treatment. After the end of this intervention taping was applied on TMJ (y shape for improved range of motion) patient education and self-assessment regarding the symptoms were considered.

### Baseline treatment

Baseline treatment was the same in both groups 1 and 2. Both groups received ultrasound before doing interventions and tapping after the intervention. Y-shape tape was applied on the masseter muscle for 4 days after the interventions. The duration of the session was 30 min, 2 sessions per week, a total of 8 sessions in 4 weeks. Measurement was calculated at baseline and the end of the treatment. Patient education and guidelines were given regarding the aggravating symptoms.

### Outcome measures

Numeric Pain Rating Scale to measure pain.

Maximum mouth opening scale (inter-incisal Rural) to determine the range of motion [24].

Jaw functional limitation scale, 20 items to check functional ability [22].

### Statistical analysis

The data was analyzed using SPSS for Windows software, version 25. Statistical significance was set at *P* = 0.05. First, the Normality of the data was checked by using Shapiro Wilk test. Parametric and non-parametric tests were applied based on normality tests.

## Results

The data was homogeneous at baseline. The Shapiro-Wilk test shows parametric data (*p* > 0.05). An independent t-test was applied between the group analysis and a paired t-test for within-group analysis. Analysis of 24 participants was done and there was no dropout in this study. Table 1 shows the demographics of participants including age, weight, height and body mass index (BMI). Table 2 shows the between-group and within-group comparison NPRS, Global Score and Jaw functional limitation scale. Both tests (paired and independent t-tests) show that NPRS, Global Score and Jaw Functional Limitation Scale of both groups show significant results (*p* < 0.05) with large effect size. Table 3 shows between-group comparison and within-group comparison of mouth opening, right and left lateral deviation. The post-isometric relaxation group shows more significant results (*p* < 0.05) than the other Bowen group with large effect size. Within-group comparison shows that both groups show significant results (*p* < 0.05) with large effect size.

**Table 1:** Demographic Characteristics

	Group 1	Group 2	*p*-value
Age (years)	33.33 ± 7.47	34.91 ± 7.86	0.618
Weight (kg)	71.7 ± 15.04	70.33 ± 14.25	0.815
Height (m)	1.65 ± 0.63	1.75 ± 0.17	0.360
BMI (kg/m^2^)	26.70 ± 5.07	23.60 ± 4.85	0.140

[Abbreviations: BMI = Body Mass Index]

**Table 2:** Across and Within Group Analysis of NPRS, Global Score and Jaw Function Limitation Scale

NPRS	Group 1	Group 2	Mean difference (Independent t-test)	*p*-value (Independent t-test)	Cohen’s d (effect size)
Pre-Value	6.67 ± 0.65	6.17 ± 0.83	0.5	0.116	0.67
Post-Value	1.83 ± 0.58	2.41 ± 0.67	-0.58	0.032	0.93
Mean difference (paired t-test)	4.84	3.76			
*p*-value (paired t-test)	0.00	0.00			
Cohen’s d (effect size)	7.93	5.08			
**Global Score**	**Group 1**	**Group 2**	**Mean difference** **(Independent t-test)**	***p-value*** **(Independent t-test)**	**Cohen’s d (effect size)**
Pre-Value	4.75 ± 0.88	4.65 ± 0.72	0.1	0.21	0.12
Post-Value	1.73 ± 0.42	2.90 ± 0.45	-1.17	0.00	2.72
Mean difference (paired t-test)	20.1	10.5			
*p*-value(paired t-test)	0.00	0.00			
Cohen’s d (effect size)	29.55	17.79			
**Jaw Functional Limitation Scale**	**Group 1**	**Group 2**	**Mean difference** **(Independent t-test)**	***p-value*** **(Independent t-test)**	**Cohen’s d (effect size)**
Pre-Value	86.91 ± 17.65	89.07 ± 15.95	-2.16	0.075	0.12
Post-Value	30.25 ± 7.72	53.66 ± 10.40	-23.41	0.000	2.55
Mean difference (paired t-test)	56.66	35.41			
*p*-value (paired t-test)	0.00	0.00			
Cohen’s d (effect size)	4.1	1.92			

[Abbreviation: NPRS = Numeric Pain Rating Scale]

**Table 3:** Across and Within Group Analysis of Mouth Opening, Right and Left Lateral Deviation

Mouth Opening	Group 1	Group 2	Mean difference (Independent t-test)	*p*-value (Independent t-test)	Cohen’s d (effect size)
Pre-Value	30.90 ± 4.10	32.20 ± 3.38	-1.3	0.458	0.34
Post-Value	56.20 ± 5.48	45.52 ± 5.20	10.68	0.00	2
Mean difference (paired t-test)	-25.3	-13.32			
*p*-value (paired t-test)	0.00	0.00			
Cohen’s d (effect size)	5.23	3.04			
**Right Lateral Deviation**	**Group 1**	**Group 2**	**Mean difference** **(Independent t-test)**	***p-value*** **(Independent t-test)**	**Cohen’s d (effect size)**
Pre-Value	19.08 ± 3.17	16.83 ± 2.51	2.25	0.068	0.78
Post-Value	26.42 ± 3.98	20.58 ± 2.35	5.84	0.00	1.79
Mean difference (paired t-test)	-7.34	-3.75			
*p*-value (paired t-test)	0.052	0.00			
Cohen’s d (effect size)	2.04	1.34			
**Left Lateral Deviation**	**Group 1**	**Group 2**	**Mean difference** **(Independent t-test)**	***p-value*** **(Independent t-test)**	**Cohen’s d (effect size)**
Pre-Value	18.58 ± 4.35	16.41 ± 9.25	2.17	0.055	0.30
Post-Value	26.50 ± 3.50	20.50 ± 3.00	6.00	0.005	1.84
Mean difference (paired t-test)	-7.92	-4.09			
*p*-value (paired t-test)	0.00	0.00			
Cohen’s d (effect size)	2.01	0.59			

## Discussion

The current study aimed to determine the effects of the post-isometric relaxation technique and Bowen’s therapy on pain, range of motion and functional activities in patients with TMJ disorder. In this clinical trial, there were two groups, Group 1 (post-isometric relaxation) and Group 2 (Bowen’s therapy) along with ultrasound and tapping. 24 subjects participated in this study and received the selected intervention twice a week for four weeks. Findings of the present study depicted that functional activity i.e. (mastication, vertical mobility, verbal and non-verbal expression communications) improved to a greater extent in the PIR group as compared to Bowen’s group due to higher mean clinical difference value (3-point mastication, 2 point vertical mobility, 5 point verbal and non-verbal expressions). Although both techniques showed clinical results with large effect size (*p*-value < 0.05) but according to the study hypothesis PIR was more effective than Bowen’s therapy.

The current study promotes the clinician’s skills or ability to deal with the TMDs patient. Therapists can use both techniques to reduce the disease effects with betterment in patient’s daily functional activities and enhance the quality of life. This study improves the knowledgeable practice guidelines in the community with new regimes and novel ideas for dealing with the temporomandibular joint by using Bowen’s therapy for the TMDs. If we look at the previous studies on such topics, post-isometric relaxation (MET) was found in the literature with beneficial effects on TMDs [25–27]. However, Bowen’s therapy was also proven as an effective treatment for different soft tissues and musculoskeletal issues [28–31].

The same results were demonstrated in earlier research by Parth Trivedi and Preeti Bhat, who found that while myofascial release and MET both significantly improved pain, range of motion, and functional outcomes, muscle energy technique had a greater impact on pain and mouth range of motion (10 mm) than MFR [21]. In the current study, outcomes for the numeric pain rating scale showed better effects with higher mean reduction value for the post-isometric group than the Bowen’s group, statistically significant results showed minimal clinically important difference (MCID) value (2 points) in the PIR group. These findings were consistent with earlier research on the effects of MET and an orthotic splint. According to MCID, the study findings were statistically significant for all four groups, the MET (post-isometric) group had the greater impact on pain reduction and maximum mouth opening (3 months of treatment) [32].

A study conducted by Magdalena Gebska, showed that massage and post-isometric relaxation of the masseter muscle improved mandibular movements (range of motions) in patients with TMDs while no significant results were seen in pain alleviation. Only female patients between the ages of 20 and 45 were included in the treatment and control groups [25].

In the current investigation, both groups’ maximal mouth opening pre and post-treatment values showed a substantial improvement, however, the post-isometric group displayed more extensive effects with only a small clinical difference (10 mm). In a prior trial conducted in 2021, patients with temporomandibular joint disease were given the MET for pain relief and maximum mouth opening (MMO). Every week MMO value was noted and the total result was taken into account during the fourth week of the session. With an MCID mean difference of 25.3 from the post-treatment mean difference, the mouth-opening score significantly increased in the MET group. MET approach reduces excessive activity by stimulating the Golgi tendon organs and muscle spindles. Muscle relaxation results from stretching the muscle fibres, which activate the Golgi tendon receptors, which have an inhibitory impact on muscle tension and increase the range of motion [26]. The present study showed the results were statistically significant (*p* < 0.05) in all outcome measures of both groups PIR and Bowen’s with large effect size. The same results were seen in a previous study conducted in a first randomized controlled trial on Bowen‘s therapy for chronic pain relief. These study results found that Bowen’s therapy provided a short period of relief for pain but the effect was not seen in long periods of pain and functional activity. Bowen‘s therapy mechanism was not acting like a nociceptive procedure [17].

The earlier study done by Malgorzata Pihut, Elzbieta in 2022 found physio-therapeutic effects on temporomandibular joint disorder patients adolescents age-wise. Manual therapy (PIR) and kinesiotherapy with massage were received for group 1 and group 2 respectively. The results showed that there was a statistical difference for pain but greater effects for functional outcomes were found in the kinesiotherapy group [27]. In contrast, the present study showed that both techniques post-isometric relaxation and Bowen‘s therapy have similar results for pain, range of motion and functional activity after the treatment but greater mean differences and larger effect size (*p* < 0.05) were seen in the post-isometric relaxation group.

The strength of this study was that PIR was compared with Bowen’s therapy and it was introduced in a trial study to treat the TMDs (with Bowen’s therapy). Bowen’s therapy showed the clinical effects so it can also be used for this purpose (TMDs).

In this study, effects were not categorized according to the chronicity of the condition. The study was not constrained by the potential psychological component, which interfered with the patient’s ability to execute, response. No long-term effects and follow-ups were considered in this study. The study duration was short and in future studies, the study duration can be increased and follow-up should be considered for the efficacy of the interventions. Although the sample size was calculated by software to generalize results, further studies can be done with a larger sample size. We can also consider the age range as a limitation as it was wide but it can also help in generalizing the results if ages were taken in classifications with a larger sample size. It is recommended that another study should be done on patients with post-traumatic stiffness with a reduction of mouth opening. Interventions applied on TMJ could have shown better results if alignment of cervical posture was included in the study as cervical head posture varies in all patients.

## Conclusion

This study concluded that post-isometric relaxation was more effective in terms of pain, range of motions for mouth opening, right and left lateral deviations and functional activity of temporomandibular joint disorder patients. However, both PIR and Bowen’s therapy groups showed better clinical results according to minimal clinical difference values.

## Data Availability

Data will be available at a reasonable request from the corresponding author.

## Abbreviations

NPRS: Numeric Pain Rating Scale
TMD: Temporomandibular Disorder
MET: Muscle Energy Technique
PIR: Post-Isometric Relaxation
CONSORT: Consolidated Standards of Reporting Trials
